# An OMV-Based Nanovaccine Confers Safety and Protection against Pathogenic *Escherichia coli* via Both Humoral and Predominantly Th1 Immune Responses in Poultry

**DOI:** 10.3390/nano10112293

**Published:** 2020-11-20

**Authors:** Rujiu Hu, Haojing Liu, Mimi Wang, Jing Li, Hua Lin, Mingyue Liang, Yupeng Gao, Mingming Yang

**Affiliations:** 1 College of Animal Science and Technology, Northwest A&F University, No.22 Xinong Road, Yangling 712100, Shaanxi, China; hurujiu@nwsuaf.edu.cn (R.H.); lhj1995@nwsuaf.edu.cn (H.L.); wmm@nwsuaf.edu.cn (M.W.); 17208046@nwsuaf.edu.cn (H.L.); lmy20@nwsuaf.edu.cn (M.L.); 2 Department of Animal Engineering, Yangling Vocational and Technical College, No.24 Weihui Road, Yangling 712100, Shaanxi, China; lijing0916@nwafu.edu.cn

**Keywords:** avian pathogenic *Escherichia coli*, nanovaccine, outer membrane vesicles, immune response, broiler

## Abstract

Avian pathogenic *Escherichia coli* (APEC) infection in poultry causes enormous economic losses and public health risks. Bacterial outer membrane vesicles (OMVs) and nano-sized proteolipids enriched with various immunogenic molecules have gained extensive interest as novel nanovaccines against bacterial infections. In this study, after the preparation of APEC O2-derived OMVs (APEC_OMVs) using the ultracentrifugation method and characterization of them using electron microscopy and nanoparticle tracking analyses, we examined the safety and vaccination effect of APEC_OMVs in broiler chicks and investigated the underlying immunological mechanism of protection. The results showed that APEC_OMVs had membrane-enclosed structures with an average diameter of 89 nm. Vaccination with 50 μg of APEC_OMVs had no side effects and efficiently protected chicks against homologous infection. APEC_OMVs could be effectively taken up by chicken macrophages and activated innate immune responses in macrophages in vitro. APEC_OMV vaccination significantly improved activities of serum non-specific immune factors, enhanced the specific antibody response and promoted the proliferation of splenic and peripheral blood lymphocytes in response to mitogen. Furthermore, APEC_OMVs also elicited a predominantly IFN-γ-mediated Th1 response in splenic lymphocytes. Our data revealed the involvement of both non-specific immune responses and specific antibody and cytokine responses in the APEC_OMV-mediated protection, providing broader knowledge for the development of multivalent APEC_OMV-based nanovaccine with high safety and efficacy in the future.

## 1. Introduction

Avian pathogenic *Escherichia coli* (APEC) is one of the major pathogens that have been recognized as serious threats to the global poultry industry [1,2,3]. APEC causes a variety of local and systemic diseases in many avian species, such as colibacillosis in broiler chickens [2,4]. Chicken colibacillosis is characterized by high morbidity and mortality, leading to substantial economic losses every year in the poultry industry worldwide [5]. In commercial production, antibiotic regimens are used as a common measure to control APEC. However, the prevalence of multidrug-resistant APEC caused by the extensive usage of antibiotics has attracted significant concerns. In many countries, antibiotics have been banned in the animal food industry [6]. Furthermore, drug residues and transfers of resistant genes through poultry products are becoming severe threats to public health [7]. Therefore, it is necessary to explore novel preventive approaches. To date, the use of effective vaccines is recognized as an important way to control APEC infections in today’s large-scale poultry industry [8,9].

Many attempts have been made to develop various biological materials as vaccine candidates against APEC infections. Some cell-wall components and virulence factors from APEC strains, such as lipopolysaccharide (LPS), outer membrane proteins, siderophore receptor protein, fimbriae and adhesins, have been shown to induce protective immunity against their corresponding serotypes [8,10,11]. Accordingly, numerous APEC vaccines, mainly inactivated, live-attenuated and subunit vaccines, have been developed for commercial use. Although these vaccines have been proven to be effective, there are still some drawbacks in practical application [8]. Inactivated vaccines are prepared by inactivating the live whole-bacteria with heat or chemicals, which can provide short-term protection against the homologous serogroups only [8]; live-attenuated bacteria vaccines may cause public safety concerns due to their potential risk of bacterial spread [9]. Subunit or recombinant vaccines, mainly including iron regulated outer membrane proteins-based vaccines, fimbriae-based vaccines and increased serum survival protein-based vaccines, could induce better protective immunity against heterologous challenges than inactivated vaccines, but they are rarely used in practice because of their limitations, such as unstable efficacy and the requirement of strong adjuvants [8,9]. Additionally, APEC isolates commonly have a variety of O serogroups (according to the O-antigens); and three main serogroups, including O1, O2 and O78, are frequently associated with disease formation in poultry farms, which can cause over 80% of chicken colibacillosis cases [2,4,8]. It may be difficult to achieve better prevention efficiency for APEC multi-serogroups using these above-mentioned vaccines. Therefore, it is still necessary to develop new vaccine candidates with both higher safety and better efficacy.

Almost all domains of life, including bacteria, archaea and eukaryotes, can secrete nanosized membrane vesicles during their normal growth [12]. These nanovesicles released by Gram-negative bacteria originate from the outer membrane of the cell envelope, and thus are also termed outer membrane vesicles (OMVs) [13]. Biochemical and proteomic analyses have shown that OMVs are naturally enriched with many bioactive molecules of the parental bacteria, including outer membrane proteins and lipids, periplasmic proteins, polysaccharides, nucleic acids (DNA and RNA) and virulence-associated factors [14,15,16,17]. Bacterial OMVs have been proven to play important roles in host–bacteria interactions, such as mediating pathogenesis, enhancing bacterial survival under various environmental stress conditions and modulating host immunity [18,19]. Due to the unique structural and immunological properties, such as biocompatible nanometer-scale structure, and the feature of being genetically modified and naturally carrying both adjuvants and multiple antigens, OMVs are generally considered to be emerging candidates for drug delivery platforms and nanovaccines [20,21,22,23]. Numerous studies have demonstrated that OMVs secreted by a variety of pathogens, such as *Neisseria meningitidis* [24], *Klebsiella pneumoniae* [25], *Vibrio cholerae* [26] *Bordetella pertussis* [27], *Salmonella* [28] and *Staphylococcus aureus* [29], can elicit protection against the corresponding bacterial infections in mice. Moreover, some studies have shown that immunization with OMVs can provide broad cross-protection against heterologous serogroups [30,31,32].

Although extensive studies have revealed that OMVs secreted by pathogenic *E. coli* species can induce protective immunity in mouse models, very few investigations have focused on whether OMVs produced by APEC (APEC_OMVs) have the potential to be developed as a novel vaccine candidates in chickens [21,33,34]. Recently, Wang and colleagues have revealed that vaccination with APEC O78-derived OMVs can protect broiler chickens against homologous infection, suggesting the potential of APEC-derived OMVs as APEC vaccine candidates [35]. Wang’s report only characterized the specific antibody responses induced by OMVs; however, the safety of APEC_OMVs and the protective mechanisms involving both innate and specific cellular immunity have not been clearly identified.

In this study, we isolated and purified APEC_OMVs from a clinical APEC O2 strain, a major APEC serogroup causing chicken colibacillosis. Compared with Wang’s study, the present study investigated the immunogenicity of APEC_OMVs in a broiler chick model using both in vitro and in vivo experiments, including innate immune responses in chicken macrophages; non-specific immune factor activities and specific antibody responses in the serum; and lymphocyte proliferation and cytokine responses in splenic lymphocytes. Moreover, we also evaluated the adverse effects of APEC_OMVs and estimated the window dose between effectiveness and toxicity for APEC_OMVs. Our work reveals the detailed immunologic mechanisms of APEC_OMV-mediated protection, providing the basic information for the development of an effective and multivalent APEC_OMV-based nanovaccine in the future.

## 2. Materials and Methods

### 2.1. Bacterial Strain and Preparation of APEC_OMVs

A clinical APEC O2 strain from a chicken with colisepticemia (collection number CVCC1554) was purchased from China Veterinary Culture Collection Center (China Veterinary Drug Supervision Institute, Beijing, China) and used in this study. This APEC isolate was grown in Luria–Bertani (LB) broth at 37 °C. Native OMVs were isolated and purified from bacterial culture supernatant by a series of centrifugal processes, as described in our previous studies [36,37]. Briefly, the bacteria-free supernatant was collected from the culture medium in the logarithmic phase by centrifugation (12,000× *g*, 15 min, 4 °C), and filtered through a 0.45-μm membrane (Merck Millipore, Tullagreen, Carrigtwohill, Ireland) followed by ultracentrifugation (150,000× *g*, 2 h, 4 °C). After washing with sterile phosphate buffer saline (PBS; pH 7.4), the obtained APEC_OMV pellet was purified by discontinuous density centrifugation. For purification, APEC_OMVs were covered with 20% (1.127 g/mL) and 35% (1.199 g/mL) OptiPrep (Sigma, catalogue number D1556) and then subjected to ultracentrifugation (16 h, 180,000× *g*, 4 °C) [38]. The interlayer of the 20% and 35% OptiPrep containing the majority of vesicles was collected, dispersed in sterile PBS and then centrifuged (150,000× *g*, 2 h, 4 °C) to completely remove OptiPrep. The purified APEC_OMVs were uniformly dispersed in sterile PBS and any bacterial contaminations were removed by filter sterilization with a 0.45-μm membrane. The APEC_OMVs samples were stored at −80 °C for future use. The protein quantification of APEC_OMVs was performed using a TaKaRa BCA Protein Assay Kit (TaKaRa Bio, Beijing, China; catalogue number T9300A) following the manufacturer’s instructions.

### 2.2. Electron Microscopy Analysis

APEC_OMVs were visualized by scanning electron microscopy (SEM) and transmission electron microscopy (TEM). For SEM visualization, 10 μL of the purified APEC_OMVs was dropped on a 5 mm × 5 mm silicon slice, dried at 20 °C and sputter-coated with gold-palladium using an ion-sputtering coater (E-1045; Hitachi, Tokyo, Japan). The prepared nanovesicles were observed using a Field Emission Scanning Electron Microscope (S-4800, Hitachi, Tokyo, Japan). For TEM visualization, 200 μg/mL of the nanovesicles was adhered to 300-mesh copper grids for 10 min followed by negatively staining with 1% phosphotungstic acid (pH 7.2), and then viewed using FEI Tecnai™ G2 Spirit BioTWIN (FEI Company, Hillsboro, OR, USA) at 100 kV.

### 2.3. Nanoparticle Tracking Analysis

Nanoparticle tracking analysis (NTA) was performed to measure the diameter size and particle number of APEC_OMVs using an NS300 nanoparticle analyzer (Malvern, Worchestershire, UK). These nanovesicle samples were uniformly dispersed in PBS and detected with a camera level of 15. Five 60 s video records were obtained for each sample and analyzed using NTA software version 2.3. The detection threshold was set at 6.

### 2.4. Animals and Housing

Broiler chicks (Arbor Acres) were hatched from fertilized eggs in an automatic incubator (Beijing LanTianJiao Electronic Technology Co., Ltd., Beijing, China) following routine incubation procedures in a sterilized room with filtered air. All hatching eggs and the incubator were sterilized before incubation. The hatching eggs were sterilized by wiping the surface of the eggshell with 75% alcohol cotton balls before putting them into the incubator. The incubator was placed in an isolation room and the isolation room was disinfected by formaldehyde fumigation. Newly hatched chicks were housed in stainless-steel cages in sterilized rooms with filtered air, strict sanitary conditions and age-appropriate temperatures. All chicks were fed an age-appropriate commercial diet containing no antibiotic additives. Drinking water and diets were offered ad libitum. All procedures of animal experiments were approved by the Ethics Committee of Animal Care and Use at Northwest A&F University with the permit number 2018NWAFU-052.

### 2.5. Maternal Anti-APEC Antibody Levels in Broiler Chicks

The objective of this experiment was to detect the optimal age for the immunization in young broiler chicks. A total of 30 newly hatched chicks were randomly divided into 6 replicates with 5 birds per replicate. The serum samples were collected at 1, 3, 5, 7, 10, 14, 18 and 21 days of age to determine the natural anti-APEC maternal antibody levels as described below.

### 2.6. Effect of APEC_OMV Vaccination on the Growth Performance, Immune Organ Index and Blood Cell Counts

This experiment aimed to evaluate the potential adverse effects of APEC_OMVs. For vaccination procedures, a total of 120 seven-day-old broiler chicks were randomly divided into four groups. Each group contained 6 replicates with 5 birds per replicate, which were respectively immunized with 200 μL PBS (as a control) and 10, 50 and 200 μg of APEC_OMVs in 200 μL PBS via intramuscular injection into the right thigh muscle using the disposable syringe at 7 and 14 days of age. The body weight, feed intake and the number of deaths were recorded on a replicate basis and used to calculate average daily weight gain (ADWG), average daily feed intake (ADFI) and feed conversion rate (FCR) from 7 to 21 days of age. At 21 days of age (one week after the secondary vaccination), 6 chicks of each group were chosen and euthanized to collect immune organs (thymus, spleen and bursa of Fabricius) and blood samples for the determination of immune organ index and blood cell counts. The organ index was calculated based on the following formula: organ index = organ weight (g)/body weight (kg). The numbers of red blood cells (RBC) and white blood cells (WBC) were estimated by a manual hemocytometer using Natt–Herrick’s stain solution [39].

### 2.7. Effect of APEC_OMV Vaccination on the Protective Efficacy against Homologous Infection in Broiler Chicks

After the vaccination procedures, the remaining 24 chicks of each group were challenged by the air sac route with 5 × 10^8^ CFU/bird of APEC O2 recommended by the previous study at 21 days of age [10]. The survival rate of chicks in each group was calculated daily for 10 consecutive days. Blood samples were collected from PBS- and APEC_OMV-immunized chicks at 12, 24 and 36 h after bacterial challenge for the determination of bacterial loads. Blood samples were prepared by 10-fold serial dilution in sterile PBS, followed by plating on LB agar plates in triplicate. The counts of bacterial colonies under 37 °C for 12 h were recorded. Serum samples were collected from PBS- and APEC_OMV-immunized chicks at 24 h after bacterial challenge for the determination of proinflammatory cytokines interleukin (IL)-1β and IL-6 using the Chicken Interleukin 1β ELISA Kit (Cloud-Clone Corp., Houston, TX, USA; catalogue number SEA563Ga) and Chicken Interleukin 6 ELISA Kit (Cloud-Clone Corp., Houston, TX, USA; catalogue number SEA079Ga) according to the manufacturer’s instructions, respectively.

### 2.8. In Vitro Chicken Macrophage Assays

HD11 cells, a chicken macrophage cell line derived from bone marrow [40], were used in this study and cultured in the complete PRMI-1640 medium (Gibco, catalogue number 22400089) supplemented with 10% heat-inactivated fetal bovine serum (FBS; Zeta-Life, catalogue number Z7181FBS-500), 100 U/mL penicillin and 100 μg/mL streptomycin (Sigma, catalogue number P4333) in an atmosphere of 5% CO_2_ at 37 °C. APEC_OMVs (5 μg/mL) were stained with 1 μM dialkylcarbocyanine iodide (DiI; Sigma, catalogue number 42364) as described previously [41]. The DiI-labeled APEC_OMVs were co-incubated with HD11 cells (5 × 10^6^ cells/well) in a 24-well culture plate. After 4 h-incubation, the cells were collected, washed and then fixed with 4% paraformaldehyde in PBS followed by cell nucleus staining with 10 μg/mL of 4′,6-diamidino-2-phenylindole (DAPI; Sigma, catalogue number D9542). Subsequently, the samples were placed on glass slides and viewed by an Andor Revolution XD spinning-disk confocal microscope (Andor Technology, UK).

To investigate innate immune responses induced by the APEC_OMVs, HD11 cells (5 × 10^6^ cells/well) were stimulated with serial doses of APEC_OMVs (0–1000 ng/mL) in a 24-well culture plate. After a 16 h treatment, the cells were harvested for the measurement of the expression of major histocompatibility complex class II β (MHC IIβ) and cytokines tumor necrosis factor α (TNF-α) and IL-6 by quantitative real-time PCR (qRT-PCR).

### 2.9. Serum Non-Specific Immune Factor Activities

Serum samples were collected from PBS- and APEC_OMV-immunized chicks at 14 and 21 days of age (one week after the primary and secondary vaccinations, respectively) before APEC_OMV vaccination or bacterial challenge. The activities of lysozyme and superoxide dismutase (SOD) in the serum were measured using commercial assay kits (Nanjing Jiancheng Bioengineering Institute, Jiangsu, China; catalogue number A050-1-1 and A001-3-2). Serum complement 3 level was estimated using a chicken-specific ELISA kit (Cloud-Clone Corp., Houston, TX, USA, catalogue number SEA861Ga). The respiratory burst activity in blood leukocyte was estimated as described previously [42].

### 2.10. Determination of Specific Antibody Titer and Bactericidal Activity in Serum

The natural antibody levels in nonimmunized serum and the levels of APEC_OMV-reactive IgY and APEC-reactive IgY in PBS- and APEC_OMV-immunized serums were determined by the ELISA method as previously described [35]. Bacterial lysates or APEC_OMVs (200 ng/well) were used as antigens and the diluted serum samples (1:200) were used as the primary antibodies. After incubation with antigens, the primary antibodies were reacted with the secondary horseradish peroxidase-conjugated rabbit anti-chicken IgY (200 ng/mL; abcam, catalogue number ab97140) followed by termination with substrate tetramethylbenzidine (100 μL). The OD_450_ was detected using a BioTek synergy2 microplate reader (Biotek, Winooski, VT, USA).

To further evaluate serum antibody responses induced by APEC_OMVs, the ability of bacteria-killing by PBS- and APEC_OMV-immunized serum samples was estimated according to the previously described method [35]. The bacterial survival rate was calculated by comparing bacterial colony-forming unit (CFU) counts after and before the treatment with the serum. Each sample was detected in triplicate.

### 2.11. Lymphocyte Proliferation Assays

Splenic and peripheral blood lymphocytes were freshly isolated from PBS- and APEC_OMV-immunized chicks at 21 days of age (one week after the secondary vaccination) using the Chicken Splenic and Peripheral Blood Lymphocyte Isolation Kits (Solarbio, catalogue number P9120 and P8740), respectively, following the manufacturer’s instructions. These isolated lymphocytes were maintained in complete PRMI-1640 medium supplemented with 10% FBS, 100 U/mL penicillin and 100 μg/mL streptomycin in an atmosphere of 5% CO_2_ at 37 °C. Cell proliferation responses were detected by 3-(4,5-dimethylthiazol-2-yl)-2,5-diphenyl tetrazolium bromide (MTT) assay using an MTT Cell Growth Assay Kit (Sigma, catalogue number CT02) as previously described [43]. The results of the tests were expressed as proliferation index according to the formula: proliferation index = (OD_570_ of test sample-OD_570_ of the negative control)/OD_570_ of the negative control. Each sample was determined in triplicate.

### 2.12. Re-Stimulation Assay of Splenic Lymphocyte

Splenic lymphocytes were obtained and cultured as described above. These cells were adjusted to a concentration of 5 × 10^6^ cells/well in a 24-well culture plate and then re-stimulated with APEC_OMVs (5 μg/mL) for 12 h. The cells were harvested to measure the gene expressions of IFN-γ, IL-4 and IL-17A.

### 2.13. Quantitative Real-Time PCR (qRT-PCR) for mRNA Quantification

Total RNA was extracted from the cultured HD11 cells and splenic lymphocytes using a Total RNA Kit (Omega Bio-Tek, catalogue number R1034) following the manufacturer’s instructions. After examination of RNA purity and quality with an ND-1000 spectrophotometer (Nano-drop Technologies, Wilmington, Delaware), the qRT-PCR analysis was performed with One Step TB Green^®^ PrimeScript™ PLUS RT-PCR Kit (TaKaRa Bio, Beijing, China; catalogue number RR096A) on an iCycler IQ5™ Real-Time PCR Detection System (Bio-Rad, Hercules, CA, USA) according to the manufacturer’s instructions. The primer sequences of the target genes and a housekeeping gene (β-actin) are shown in Supplementary Table S1. Triplicate qRT-PCR reactions for each sample were conducted with the following protocol: 95 °C for 1 min; 40 cycles of 95 °C for 15 s and 60 °C for 30 s, 72 °C for 30 s; 72 °C for 10 min. Relative mRNA expression was calculated using the 2^−ΔΔCt^ method as described previously [44], and expressed as the fold-change relative to the control, which was normalized to 1.

### 2.14. Statistical Analysis

Experimental data were shown as mean ± standard error (S.E.). Data were analyzed by Graph Pad Prism software 5.0 (San Diego, CA, USA). The analysis of differences between the two groups was performed by Student’s *t*-test. The analysis of differences among greater than two groups was performed by one-way ANOVA analysis with the Newman–Keuls post-test. The survival data after bacterial infection were analyzed by the log-rank test. Statistical significance was declared at *P* < 0.05.

## 3. Results

### 3.1. Characterization of APEC_OMVs

**Figure 1:**
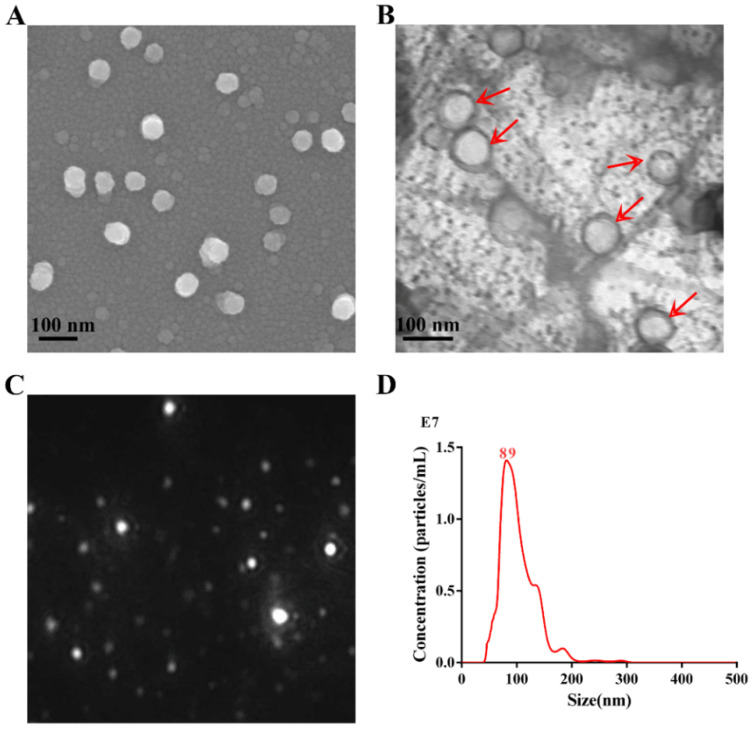
Characterization of outer membrane vesicles secreted by avian pathogenic *E. coli* O2 (APEC_OMVs). (**A**) The purified APEC_OMVs were viewed with a scanning electron microscope. (**B**) After negative staining with 1% phosphotungstic acid, APEC_OMVs were visualized by transmission electron microscopy. The red arrows indicate several visible APEC_OMVs. (**C**) The image from the movie captured using a SCMOS camera of Malven NTA 3.0 when APEC_OMVs were characterized by nanoparticle tracking analysis (NTA). (**D**) Concentration and size distribution of APEC_OMVs determined by NTA.

APEC_OMVs were obtained from the culture supernatant of APEC O2 strain using a series of centrifugation procedures and then purified using the density gradient centrifugation method. The morphology and integrity of APEC_OMVs were detected by SEM (Figure 1A) and TEM (Figure 1B). The results showed that these vesicles were intact nanosized structures with spherical morphology, and the majority of these nanovesicles ranged from 50 to 150 nm in diameter. Typical results characterized by NTA are shown in Figure 1C,D. The diameter of APEC_OMVs peaked at 89 nm, which is in accordance with the results of electron microscopy analyses and the previously determined sizes of bacterial OMVs [34].

### 3.2. Natural Antibody Levels in Nonimmunized Chicks

**Figure 2:**
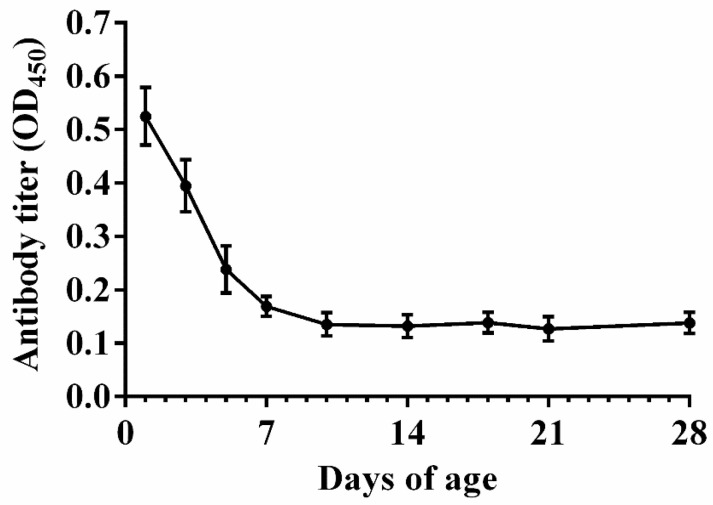
Natural anti-APEC maternal antibody levels in the serum collected from commercial broiler chicks during the 1–28 days of age (n = 5). Data are presented as mean ± SE.

As shown in Figure 2, a high antibody level was observed in the serum before 5 days of age. The antibody titer dropped to a low level at 7 days of age and remained stable thereafter. These findings suggested that the optimal age of vaccination should not be earlier than 7 days of age.

### 3.3. Effect of APEC_OMVs Vaccination on the Growth Performance, Immune Organ Index and Blood Cell Counts

As shown in Table 1, vaccination with 10 and 50 μg of APEC_OMVs had no significant impacts on ADFI, ADWG, FCR, immune organ index or WBC and RBC counts. However, vaccination with 200 μg of APEC_OMVs significantly reduced ADFI and ADWG, and increased FCR and WBC count, suggesting that this vaccination dose can have adverse effects on chicks. The immune organ indexes were slightly improved in all APEC_OMV-immunized groups compared with the control group, but an increase in the dose of APEC_OMVs to 200 µg had no statistically significant effect on the immune organ indexes. No chicks died in all groups during the vaccination period (data not shown). These results demonstrated that vaccination with an appropriate dose of APEC_OMVs was safe, while high doses of APEC_OMVs could be toxic.

### 3.4. Vaccination with APEC_OMVs Was Protective against Homologous Infection in Broiler Chicks

**Figure 3:**
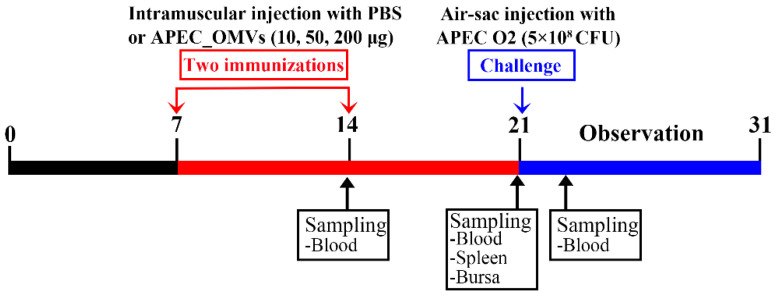
Timeline of APEC_OMV vaccination and bacterial challenge in broiler chick experiments. Four groups of broiler chicks (n = 30; 6 replicates per group with 5 birds per replicate) were intramuscularly immunized with PBS (as a control) and various doses of APEC_OMVs at 7 and 14 days of age, respectively, and challenged by the air sac route at 21 days of age. Blood and spleen samples were collected from PBS- and APEC_OMV-immunized chicks at indicated times.

**Figure 4:**
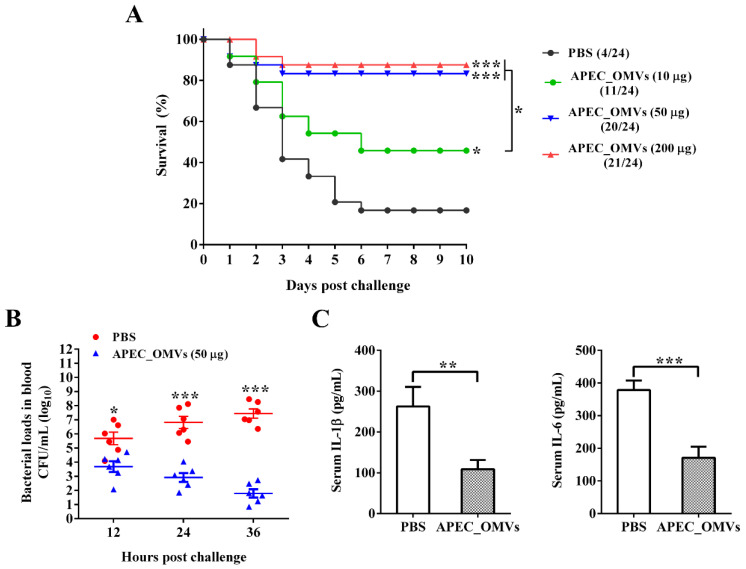
Protective efficacy conferred by APEC_OMV vaccination against homologous challenge in broiler chicks. (**A**) Survival rates of PBS- and APEC_OMV-immunized birds after bacterial challenge (n = 24). The number of surviving chicks in each group was recorded daily for 10 consecutive days after bacterial challenge. (**B**) Bacterial loads in peripheral blood collected from PBS- and APEC_OMV (50 μg)-immunized chicks at indicated times after bacterial challenge (n = 6). (**C**) The production of proinflammatory cytokines (IL-1β and IL-6) in the serum collected from PBS- and APEC_OMV (50 μg)-immunized chicks at 22 days of age (24 h after bacterial challenge) (n = 6). Data are presented as mean ± SE. * *p* < 0.05; ** *p* < 0.01; *** *p* < 0.001, versus the respective control.

The procedures for the vaccination and bacterial challenge are shown in Figure 3. As shown in Figure 4A, only 16.7% of chicks in the PBS-immunized group survived 10 days after the bacterial infection. The survival rates of 45.8% and 83.3% were observed for groups immunized with 10 and 50 μg of APEC_OMVs, respectively, which were significantly higher than those in the PBS-immunized group. However, an increase of the vaccination dose to 200 μg did not result in significant improvement of protective efficacy. Together with the results of experiment 2, the dose of 50 μg was selected as the final dosage for the following analyses. To further confirm the protective efficacy conferred by APEC_OMVs, bacterial loads in blood samples and proinflammatory cytokine production in serum samples were determined after bacterial challenge. As illustrated in Figure 4B, the APEC_OMV-immunized group showed significantly lower bacterial counts in the blood after 24 h post-infection compared with the control group, indicating that effective clearance of bacteria was induced by APEC_OMVs. Additionally, the levels of proinflammatory cytokines IL-1β and IL-6 in the APEC_OMV-immunized serum were significantly lower than those in the PBS-immunized serum (Figure 4C). These results revealed that vaccination with APEC_OMVs could reduce bacterial loads and proinflammatory cytokine production, and thus provided protection against homologous challenge.

### 3.5. APEC_OMVs Activated Innate Immune Responses In Vitro

**Figure 5:**
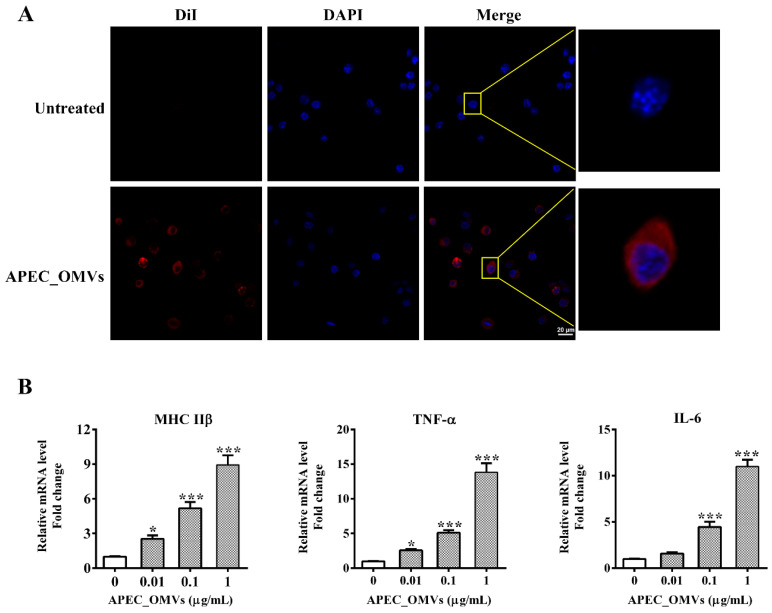
Innate immune responses induced by APEC_OMVs in chicken macrophages in vitro. (**A**) Visualization of internalization of APEC_OMVs by chicken HD11 macrophages using a confocal microscope. Row 1 was a non-stimulated treatment. Row 2 was stimulated with 5 μg/mL of DiI-labeled APEC_OMVs (red signal) for 6 h at 37 °C followed by cell nucleus staining with 10 μg/mL of DAPI (blue signal). (**B**) qRT-PCR analysis for the expression of MHC IIβ, TNF-α and IL-6 in HD11 cells stimulated with various concentrations of APEC_OMVs for 16 h. Results are representatives of three independent experiments and expressed as mean ± SE. * *p* < 0.05; ** *p* < 0.01; *** *p* < 0.001, versus the control.

Macrophages are not only important players in the clearance of pathogens but also function as antigen-presenting cells (APCs) to recognize and process foreign antigens, linking the innate and adaptive immune responses. We first examined whether chicken HD11 macrophages recognize and respond to APEC_OMVs in vitro. After co-incubation with HD11 cells for 4 h, these DiI-labeled vesicles (red signals) were observed in the cytoplasms of these cells, suggesting that APEC_OMVs were internalized by HD11 cells (Figure 5A). Furthermore, stimulation with APEC_OMVs dramatically enhanced the expression of MHC IIβ and cytokines TNF-α and IL-6 in a dose-dependent manner (Figure 5B). These results indicated that APEC_OMVs could provoke innate immune responses in APCs, which can activate T cell responses.

### 3.6. Vaccination with APEC_OMVs Improved Serum Non-Specific Immune Factor Activities

**Figure 6:**
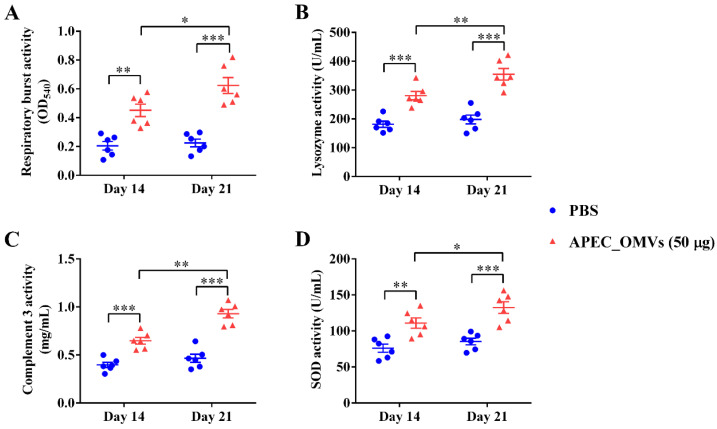
Effects of APEC_OMV vaccination on non-specific immune factor activities in the serum. Serum samples were collected from PBS- and APEC_OMV (50 μg)-immunized chicks at 14 and 21 days of age (one week after the primary and secondary immunization, respectively) before the secondary APEC_OMV immunization or bacterial challenge to determine the activities of the following immune factors: (**A**) lysozyme; (**B**) complement 3; (**C**) respiratory burst; (**D**) SOD. Data are presented as mean ± SE (n = 6). * *p* < 0.05; ** *p* < 0.01; *** *p* < 0.001.

We next evaluated the vaccination effect of APEC_OMVs on the serum non-specific immune factor activities. As shown in Figure 6, vaccination with 50 μg of APEC_OMVs significantly improved the lysozyme, complement 3, respiratory burst and SOD activities after both primary and secondary vaccinations. Moreover, the activity of these non-specific immune factors was significantly higher after the secondary booster vaccination than after the primary vaccination.

### 3.7. APEC_OMV-Induced Protection Was Associated with Elevated Antibody Responses

**Figure 7:**
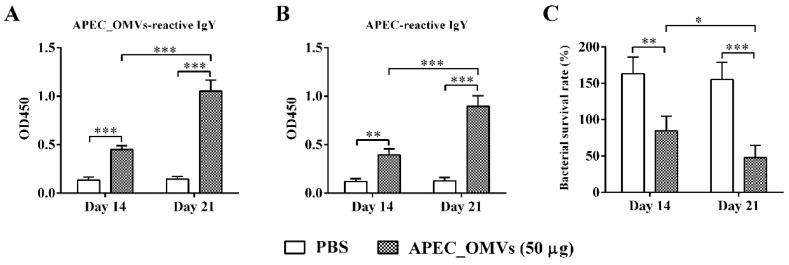
Specific IgY levels and bactericidal activities of serum samples from APEC_OMV-immunized chicks. Serum samples were collected from PBS- and APEC_OMV (50 μg)-immunized chicks at 14 and 21 days of age (one week after primary and secondary vaccination, respectively) before the secondary APEC_OMV vaccination or bacterial challenge. The production of APEC_OMV-reactive IgY (**A**) and APEC-reactive IgY (**B**) was estimated by ELISA assays. (**C**) Bacteria killing assay by the APEC_OMV-immunized serum. The APEC O2 strain was incubated with PBS- and APEC_OMV (50 μg)-immunized serum samples at 37 °C for 1 h and then the bacterial survival rate was measured. Data are presented as mean ± SE (n = 6). * *p* < 0.05; ** *p* < 0.01; *** *p* < 0.001.

To identify adaptive immune responses involved in APEC_OMV-induced protection, we first detected the specific antibody responses one week after the primary and secondary vaccinations. The levels of APEC_OMV-reactive IgY and APEC-reactive IgY were significantly elevated when chicks were immunized with APEC_OMVs (Figure 7A,B). The secondary booster vaccination significantly enhanced the antibody responses compared to the primary vaccination. Furthermore, APEC_OMV-immunized serum samples showed higher bactericidal activities after both primary and secondary vaccinations compared with PBS-immunized serum samples (Figure 7C).

### 3.8. Vaccination with APEC_OMVs Induced Lymphocyte Proliferation and a Predominant Th1 Response

**Figure 8:**
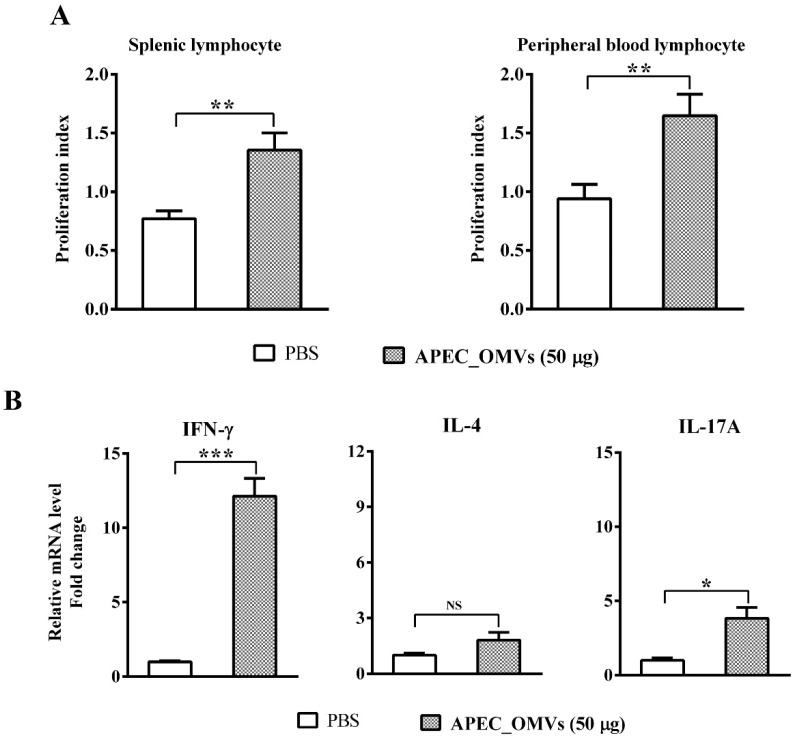
Lymphocyte proliferation and cytokine responses induced by APEC_OMVs. At 21 days of age (one week after secondary vaccination) before the bacterial challenge, splenic lymphocytes and peripheral blood lymphocytes were isolated from PBS- and APEC_OMV (50 μg)-immunized chicks. (**A**) Proliferation indexes of MTT assays for the splenic lymphocytes and peripheral blood lymphocytes re-stimulated with mitogen (ConA). (**B**) Analysis of mRNA expression of Th1 cytokine (IFN-γ), Th2 cytokine (IL-4) and Th 17 cytokine (IL-17) in the splenic lymphocytes re-stimulated with APEC_OMVs (5 μg/mL) for 24 h. Data are presented as mean ± SE. * *p* < 0.05; ** *p* < 0.01; *** *p* < 0.001; NS, not significant.

We finally identified cellular responses associated with protection conferred by APEC_OMVs. As shown in Figure 8A, APEC_OMV vaccination significantly enhanced the proliferation of both spleen lymphocytes and peripheral blood lymphocytes in response to mitogen. To determine which T-cell responses were involved in APEC_OMV-induced protection, spleen lymphocytes were isolated from the immunized chicks one week after the secondary vaccination and re-stimulated with APEC_OMVs. The results showed that re-stimulation with APEC_OMVs significantly upregulated the expression of IFN-γ (a representative Th1 cytokine) and IL-17A (a representative Th17 cytokine); the expression of IL-4 (a representative Th2 cytokine) remained unchanged between PBS- and APEC_OMV-immunized groups. Meanwhile, the degree of IFN-γ upregulation (over 12-fold change compared with the control) was much higher than that of IL-17A upregulation (only 3.8-fold change compared with the control), suggesting that IFN-γ-mediated Th1 response may play a predominant role.

## 4. Discussion

Vaccination has proved to be the most practical and effective strategy to control bacterial infections. Bacterial OMVs are becoming increasingly attractive as effective immune-stimulating materials for the development of novel vaccines and adjuvants [20]. Avian colibacillosis, caused by APEC strains, is one of the most severe diseases leading to large economic losses in the global poultry industry [45]. In the present study, we confirmed that vaccination with 50 μg of APEC_OMVs showed no adverse effects and effectively protected chicks against homologous APEC infection by reducing bacterial loads and proinflammatory cytokine production. These protective effects were mediated through activations of both innate and adaptive immune responses elicited by APEC_OMVs, which mainly included serum non-specific immune factors, a specific antibody-mediated humoral immune response and an IFN-γ-mediated cellular immune response.

OMV-based vaccines hold several advantages over the current inactivated and attenuated live APEC vaccines. First, a large number of studies and reviews have revealed that OMVs are membranous vesicles with nanoscale sizes in the range of 20–250 nm, which enables them highly biocompatible and capable of delivering interior molecules in concentrated and protected forms [13,46,47]. Consistent with these previous results, electron microscopic and NTA analyses in our work showed that APEC_OMVs were intact spherical bilayer nanoparticles with a mean size of 89 nm in diameter. These structural characteristics allow OMVs to be more effectively delivered throughout intracellular compartments and efficiently taken-up by APCs. Second, OMVs carry various immune-stimulating molecules from the outer membrane, such as LPS and outer membrane proteins and lipids [13]. Many of these components are immunogenic and act as natural adjuvants, which have the potential to be both multi-antigen carriers and vaccine adjuvants [33,34,48]. These immunological characteristics of these OMV-based vaccines provide great advantages in vaccine efficacy compared to the current single-antigen vaccines. Third, in addition to being easily prepared from natural bacteria, OMVs can be conveniently loaded with specific antigens using bioengineered bacteria [49,50]. Finally, they showed better safety within a certain dose range than attenuated live vaccines due to their nonliving and acellular features, largely reducing the risk of bacterial transmission. However, it should be noted that injection with high doses of OMVs might be toxic because they contain various virulence-associated factors. In this study, we determined that a dose of 50 μg for APEC_OMVs not only showed no side effects but also induced effective protection. Therefore, extra considerations should be made in the window between efficacy and toxicity for OMV immunization on animals and humans in the future.

Innate immunity is recognized to be the first line of host defense against pathogen-associated invasion. Previous in vitro studies have demonstrated that OMVs could activate the innate immune responses of dendritic cells and macrophages and induce the production of cytokines that regulate the adaptive immune responses [25,33]. As an essential cellular component in the innate immune system, macrophages are not only directly involved in the elimination of pathogens, but also can be used as APCs to recognize and process antigens [51]. Consistent with these findings, in this study, we observed that APEC_OMVs were effectively taken-up by the chicken HD11 macrophage cell line in vitro and enhanced the expression of MHC IIβ, TNF-α and IL-6. MHC IIβ, mainly expressed in APCs, can assist these cells in presenting exogenous antigen to CD4 T cells, which can activate specific B- and T-cell immune responses [52]. IL-6 is known to induce adaptive immune responses in mammalian Th17 and play an important role in fighting bacterial invasion [33]. It is important to note, however, that these results were derived from a macrophage cell line, and future studies will be needed to investigate whether similar results are expected from primary cells. Additionally, non-specific immune factors in serum are essential for the elimination of pathogens. Lysozyme plays an essential role in the host’s innate immune system against bacterial challenge by cleaving cell wall peptidoglycan [53]. The complement molecule is an important innate immune component that can initiate innate responses and modulate adaptive immune responses [54]. Respiratory burst is an oxygen-dependent mechanism by which neutrophils kill invading pathogens [55]. SOD can effectively alleviate inflammatory responses by reducing oxidative stress during bacterial infection [56]. The activities of these non-specific immune factors were significantly enhanced during APEC_OMV vaccination, which may be due to the enhanced pathogen clearance accompanied by the involvement of the increased leukocytes [57].

The adaptive immune response plays a very important role in host defense against bacterial infections. Specific antibody and T-cell immune responses induced by bacterial OMVs have been demonstrated in both in vitro and in vivo mouse studies [25,33,58]. However, it is unclear which immune response plays a major role. Some studies have shown that antibody-mediated humoral immunity is the most important factor [26], while other studies have suggested that cellular immunity mediated by cytokines, especially IFN-γ and IL-17, is essential for the protection [25]. In the current study, the ELISA results showed that vaccination with APEC_OMVs could elevate the production of anti-APEC_OMVs and anti-APEC antibodies in the APEC_OMV-immunized serum. These enhanced responses of specific antibody induced by APEC_OMVs were also confirmed by bactericidal activity assays. These findings are consistent with the recent study showing that OMVs derived from APEC O78 induced similar protective efficacy in an antibody-dependent manner [35]. However, cytokine-mediated cell responses to APEC_OMV vaccination have not previously been determined. Previous studies performed in mice have indicated that the IFN-γ-mediated Th1 response plays an important role in the protection against bacterial infections by enhancing the bactericidal activity of phagocytes [33,59]. Moreover, the protective effect of vaccines against bacterial challenge requires Th17-mediated immunity by promoting neutrophil recruitment to the site of infection [60]. Meanwhile, our study indicated that APEC_OMVs promoted splenic and peripheral blood lymphocyte proliferation during vaccination. We further identified that APEC_OMVs activated the expression of IFN-γ and IL-17A but not IL-4 in splenic lymphocytes. These results are in accordance with the previous study performed in mice with pathogenic *E. coli*-derived OMVs [33]. It is worth noting that the upregulation of IFN-γ induced by APEC_OMVs was visibly higher than that of IL-17, implying that the IFN-γ-mediated Th1 response may play a dominant role. However, at present, we could not rule out whether other cell types, such as NK cells and γ-δ T cells, are involved in the protection because these cells can also produce IFN-γ and IL-17. The exact cell responses to APEC_OMVs require further exploration. Taken together, although further study is needed, our present work reveals that vaccination with APEC_OMVs induces protective immunity against homologous bacterial infection mainly through the induction of specific antibody and IFN-γ-mediated immune responses.

APEC_OMV-induced protection was well-evidenced in this study, but it is challenging to identify which specific molecules are most essential because APEC_OMVs contain a variety of immunogenic components. Many reviews have illustrated that protein is the most important component of bacterial OMVs and mediates multiple functions [13,61]. Large-scale proteomic studies of various Gram-positive bacteria-derived OMVs have indicated that outer membrane proteins account for the majority of vesicular proteins [14,62]. Many outer membrane proteins have been proved to contribute to bacterial pathogenesis and are used as protective antigens to induce effective protection against bacterial infections [63,64]. Several outer membrane proteins with high abundance, mainly OmpA, OmpX and OmpW, are commonly found in OMVs secreted by APEC strains and other *E. coli* and can elicit strong protective immunity [35,65,66]. These conserved outer membrane proteins give APEC_OMVs the ability to confer a certain degree of cross-protection. The cross-protective effects of several pathogens-derived OMVs have been demonstrated in mice [30,32]. Further investigations are required to confirm whether APEC_OMVs protect against heterologous infections. Additionally, OMVs carry various non-protein antigens, such as LPS, which also participate in the APEC_OMV-induced protective immunity [61]. It is the combination of these diverse antigens that give OMVs their extensive immunogenicity.

Although our present study may be useful to developing a new APEC vaccine, there remain several shortcomings which shall lead us to future work. First, APEC_OMV vaccination using the intramuscular route is less likely to be feasible for practical use in large flocks. Further studies can focus on the development of oral OMV-based nanovaccines for poultry, which may be more competitive and attractive for commercial use in broilers. Second, vaccinating twice for broiler chickens can be costly compared to the currently available vaccine. Third, we did not evaluate the stability and uniformity of APEC_OMVs preparation. Fourth, we did not investigate whether APEC_OMVs could provide broad protection against heterologous challenges. It is believed that single-serogroup APEC_OMVs may not provide enough protective efficacy against chicken colibacillosis caused by multiple APEC serogroups. Therefore, it is still necessary to develop multi-serogroup APEC_OMVs or bioengineered APEC_OMVs with highly expressed heterologous antigens as broadly-protective vaccine candidates against multiple APEC serogroups in the future. Finally, some important investigations of APEC_OMVs as a vaccine candidate were not carried out. For example, antibody responses were not determined after the APEC challenge, the survival of chickens and bacterial load until slaughter age (day 42) were not monitored and the APEC lesions in the internal organs were also not investigated. Further studies, including investigations of the protective efficiency and long-term protection of APEC_OMVs, more detailed protection mechanisms associated with bacterial clearance in multiple organs, pathological changes of internal organs and cytokine responses, etc., will be considered in our future research programs. In conclusion, we revealed that vaccination with APEC_OMVs protected broiler chicks against homologous infection by enhancing bacterial clearance and reducing proinflammatory cytokine production. We further demonstrated that APEC_OMVs could activate innate immune responses in HD11 macrophages in vitro and enhanced activities of serum non-specific immune factors. We also identified that APEC_OMV vaccination could induce both specific antibody responses and a predominant IFN-γ-mediated cellular immune response. Our findings provide broader knowledge to better understand the immunological basis of APEC_OMV-mediated protection and offer a novel strategy for the development of cross-protective nanovaccines to control various APEC serogroups in the future.

## Figures and Tables

**Table 1:** Effects of various doses of APEC_OMV vaccination on the growth performance, immune organ index and blood cell counts.

Item ^2^	APEC_OMVs ^1^ (μg/bird)				SE ^3^	*P*-Value
	0	10	50	200		
**Growth Performance**						
ADFI (g/d)	64.5 ^a^	63.9 ^a^	62.6 ^a^	57.2 ^b^	1.35	0.024
ADWG (g/d)	51.3 ^a^	49.6 ^a^	49.2 ^a^	42.4 ^b^	1.40	0.003
FCR	1.26 ^b^	1.29 ^b^	1.27 ^b^	1.35 ^a^	0.013	0.031
**Immune Organ Index (g/kg body weight)**						
Thymus index	2.06	2.40	2.48	2.39	0.100	0.498
Spleen index	1.09	1.164	1.21	1.12	0.045	0.801
Bursa index	1.60	1.86	1.83	1.74	0.069	0.339
**Blood Cell Counts**						
WBC (10^3^/μL)	26.7 ^b^	28.3 ^b^	31.5 ^b^	39.3 ^a^	3.82	0.042
RBC (10^6^/μL)	2.24	1.86	1.93	1.71	0.29	0.126

^1^ APEC_OMVs = avian pathogenic *Escherichia coli* O2-derived outer membrane vesicles. ^2^ ADFI = average daily feed intake; ADWG = average daily weight gain; FCR = feed conversion rate; WBC = white blood cells; RBC = red blood cells. ^3^ SE = Standard error of the mean. Growth performance were calculated from 7 to 21 days of age; immune organ index and blood cell counts were measured at 21 days (one week after the secondary vaccination). ^a,b^ Different superscript letters in the same row indicate significant difference (*p* < 0.05).

## Supplementary Materials

The following are available online at https://www.mdpi.com/2079-4991/10/11/2293/s1, Table S1: Primers used for quantitative real-time PCR in this study.
