# Supplementary material for: An OMV-Based Nanovaccine Confers Safety and Protection against Pathogenic Escherichia coli via Both Humoral and Predominantly Th1 Immune Responses in Poultry
Source: Nanomaterials (Basel). 2020 Nov 20;10(11):2293. doi: 10.3390/nano10112293 (PMC7699605; doi:10.3390/nano10112293)
Supplement: Supplementary file 1 [file nanomaterials-10-02293-s001.pdf]

nanomaterials

Supplementary Materials

An OMV-Based Nanovaccine Confers Safety and Protection against Pathogenic *Escherichia coli* via Both Humoral and Predominantly Th1 Immune Responses in Poultry

Rujiu Hu [1], Haojing Liu [1], Mimi Wang [1], Jing Li [2], Hua Lin [1], Mingyue Liang [1], Yupeng Gao [1,*] and Mingming Yang [1,*]

[1] College of Animal Science and Technology, Northwest A&F University, No.22 Xinong Road, Yangling 712100, Shaanxi, China; hurujiu@nwsuaf.edu.cn (R.H.); lhj1995@nwsuaf.edu.cn (H.L.); wmm@nwsuaf.edu.cn (M.W.); 17208046@nwsuaf.edu.cn (H.L.); lmy20@nwsuaf.edu.cn (M.L.)

[2] Department of Animal Engineering, Yangling Vocational and Technical College, No.24 Weihui Road, Yangling 712100, Shaanxi, China; lijing0916@nwafu.edu.cn

* Correspondence: gaoyupeng@nwsuaf.edu.cn (Y.G.); ymm@nwsuaf.edu.cn (M.Y.)

Table 1. Primers used for quantitative real-time PCR in this study.

Gene	Acc. No.	Primers Sequence (5'-3')	Reference
β-actin	NM_205518	F: ACACCCACACCCCTGTGATGAA<br>R: TGCTGCTGACACCTTCACCATTC	Li *et al.*, 2018 [1]
TNF-α	JN942589.1	F: AGTGCTGTTCTATGACCGCC<br>R: CGCTCCTGACTCATAGCAGA	Li *et al.*, 2018 [1]
IL-4	NM_001007079.1	F: TCTTCCTCAACATGCGTCAG<br>R: TGGTGGAAGAAGGTACGTAGG	Li *et al.*, 2018 [1]
IL-6	NM_204628.1	F: CTCCTCGCCAATCTGAAGTC<br>R: GGCACTGAAACTCCTGGTCT	Li *et al.*, 2018 [1]
IL-17A	NM_204460.1	F: ATGTCTCCGATCCCTTATTCT<br>R: CCTTTAAGCCTGGTGCTGGAT	Min *et al.*, 2002 [2]
IFN-γ	NM_205149	F: TGATGGCGTGAAGAAGGTG<br>R: GACTGGCTCCTTTTCCTTTTG	Bolha *et al.*, 2013 [3]
MHC-IIβ	NM001318995	F: CCCTCGGCGTTCTTCTTCTAC<br>R: CCCACGTCGCTGTCGAA	Lian et al., 2010 [4]

1. Li R, Li J, Zhang S, Mi Y, Zhang. Attenuating effect of melatonin on lipopolysaccharide-induced chicken small intestine inflammation. *Poult. Sci.* **2018**, *97*, 2295–2302
2. Min WI, Lillehoj HS. Isolation and characterization of chicken interleukin-17 cDNA. *J. Interf. Cytok. Res.* **2002**, *22*, 1123–1128

3. Bolha L, Benčina D, Cizelj I, Oven I, Slavec B, Rojs OZ, Narat M. Effect of *Mycoplasma synoviae* and lentogenic Newcastle disease virus coinfection on cytokine and chemokine gene expression in chicken embryos. *Poult. Sci.* **2013**, *92*, 3134–3143
4. Lian L, Qu LJ, Zheng JX, Liu CJ, Zhang YP, Chen YM, Xu GY, Yang N. Expression profiles of genes within a subregion of chicken major histocompatibility complex B in spleen after Marek's disease virus infection. *Poult. Sci.* **2010**, *89*, 2123–2129
